# Duodenal gastrointestinal stromal tumors appear similar to pancreatic neuroendocrine tumors: A case report

**DOI:** 10.1016/j.ijscr.2018.11.011

**Published:** 2018-11-15

**Authors:** Yurie Futo, Shin Saito, Hideyo Miyato, Ai Sadatomo, Yuki Kaneko, Yoshihiko Kono, Daisuke Matsubara, Hisanaga Horie, Alan Kawarai Lefor, Naohiro Sata

**Affiliations:** a Department of Surgery, Jichi Medical University, Tochigi, Japan; b Department of Surgery, JCHO Utsunomiya Hospital, Utsunomiya, Japan; c Department of Pathology, Jichi Medical University, Tochigi, Japan

**Keywords:** GIST, gastrointestinal stromal tumor, FNA, fine-needle aspiration, NET, neuroendocrine tumor, EUS, endoscopic ultrasound, CT, computed tomography, Duodenal gastrointestinal stromal tumor, Pancreatic neuroendocrine tumor, Gastrointestinal bleeding, Case report

## Abstract

**Introduction:**

Duodenal gastrointestinal tumors (GISTs) are rare. Duodenal GISTs and pancreatic neuroendocrine tumors (NETs) may appear similar on imaging studies. GISTs arising from the second or third portions of duodenum may be incorrectly diagnosed as pancreatic NETs.

**Presentation of case:**

The patient is a 79-year-old man who was referred to our hospital with a history of tarry stools and loss of consciousness. Urgent upper digestive tract endoscopy revealed a bleeding submucosal duodenal lesion, which was controlled using endoscopic clips. Enhanced computed tomography scan showed a hyper-vascular mass 50 mm in diameter, at the pancreatic uncus. The patient underwent a pylorus-preserving pancreaticoduodenectomy. Histologically, the tumor was composed of spindle-shaped cells immunohistochemically positive for c-kit and CD34, and the lesion diagnosed as a duodenal GIST.

**Discussion:**

Duodenal GISTs often present with gastrointestinal bleeding, which can necessitate emergency surgery. Surgical resection with regional lymph node dissection is the optimal treatment for pancreatic NETs. In contrast, GISTs are generally treated with a minimal resection and without lymph node dissection. Thus, establishing the diagnosis is important in the management of these tumors. Endoscopic ultrasound-guided fine needle aspiration (EUS-FNA) is effective to establish the diagnosis of these lesions.

**Conclusion:**

A tumor located in the pancreatic head or mesenteric side of the duodenum cannot always be diagnosed based on imaging, and is ideally diagnosed histologically to guide the extent of resection. While EUS-FNA can establish the diagnosis, the complications of this procedure must be considered.

## Highlights

• Duodenal GISTs and pancreatic NETs are rare.
• Duodenal GISTs often present with gastrointestinal bleeding.
• Duodenal GISTs can be misdiagnosed as a pancreatic head mass.
• The optimal surgical procedure has not yet been established for duodenal GISTs.
• The diagnosis can be established preoperatively to guide the extent of resection.

## 1 Introduction

Gastrointestinal stromal tumors (GISTs) are believed to arise from the interstitial cells of Cajal or their precursors, located throughout the muscular wall of the gastrointestinal tract [1]. Duodenal GISTs are extremely rare and account for <5% of all GISTs [2]. Duodenal GISTs of the extraluminal type can be difficult to diagnose [3]. Endoscopic ultrasound-guided fine needle aspiration (EUS-FNA) has been reported to be useful to establish the diagnosis of mural lesions in the gastrointestinal tract and tumors of adjacent organs [4]. Duodenal GISTs generally have moderate to intense enhancement on computed tomography (CT) scan [5]. Since gastrointestinal bleeding is a common complication [6], EUS-FNA may lead to bleeding.

Pancreatic neuroendocrine tumors (NETs) can occur anywhere in the pancreas and 30–40% are nonfunctional [7]. Pancreatic NETs appear as circumscribed solid masses that displace surrounding structures and often hyperattenuating on arterial and venous phase images on CT scan [8]. Since pancreatic NETs and duodenal GISTs may have similar imaging features, these two lesions can be mis-diagnosed [9]. We present a patient with a duodenal GIST which is difficult to differentiate from a pancreatic NET. This work is reported in line with the SCARE criteria [10].

## 2 Presentation of case

**Fig. 1:**
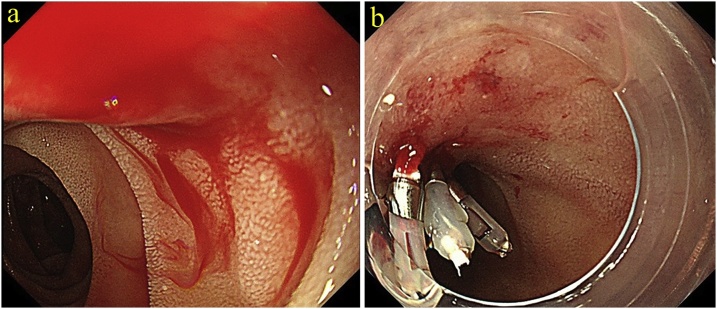
(a) Upper gastrointestinal endoscopy showed a duodenal submucosal protruding lesion which caused bleeding. (b) Gastrointestinal bleeding was controlled with endoscopic clips and the bleeding point was distal to the duodenal papilla (third portion).

**Fig. 2:**
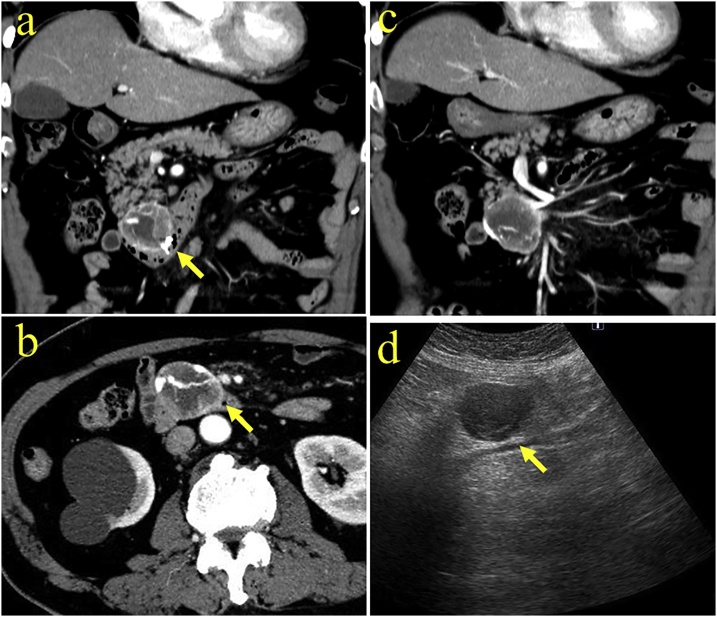
(a) Coronal contrast-enhanced computed tomography (CT) scan showed a heterogeneously enhanced 50 mm tumor in the pancreatic uncus. Arrow indicates clips used for endoscopic hematemesis. (b) Axial contrast enhanced CT scan showed a well-defined exophytic mass (arrow) accompanied by a rich network of feeding vessels passing through the mass. (c) The tumor was fed by the inferior pancreaticoduodenal artery. (d) Abdominal ultrasonography showed a 45 × 30 mm tumor in the head of the pancreas with a smooth surface and simple low-echogenicity, without pancreatic ductal dilatation (arrow).

**Fig. 3:**
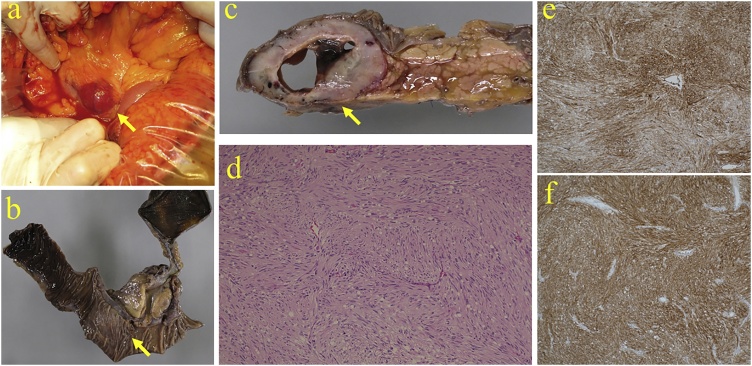
(a) At laparotomy, the tumor was found between the third portion of the duodenum and the pancreatic uncus (arrow). (b) Arrow indicates the scar after endoscopic hemostasis using clips. (c) The resected specimen showed a 45 × 28 × 50 mm submucosal tumor in the duodenum, compressed and extended to the uncus of the pancreas with central necrosis (arrow). (d) Histopathological findings showed the tumor was composed of spindle-shaped cells. (Hematoxylin-Eosin stain, 100x). (e) Immunostaining revealed that the cells were positive for c-kit. (f) The cells stained positive for CD34 (100x). Immunohistochemical study established the diagnosis of a duodenal gastrointestinal stromal tumor.

The patient is a 79-year-old Japanese man referred with complaints of tarry stool and transient loss of consciousness. He had no significant past medical history. He had conjunctival anemia and laboratory data revealed anemia (Hgb = 6.8 g/dl). The remainder of the physical examination and laboratory studies including tumor markers and hormone levels were within normal range. Upper digestive endoscopy was performed, revealing a submucosal protruding duodenal lesion with central ulceration and bleeding (Fig. 1a). The bleeding was easily controlled with endoscopic clips although the bleeding site was distal to the duodenal papilla (third portion) (Fig. 1b). An abdominal dynamic contrast-enhanced CT scan showed a 50 mm tumor in the pancreatic uncus, which was well defined and enhanced from the arterial to the venous phase (Fig. 2a, b). The tumor was supplied by the inferior pancreaticoduodenal artery (Fig. 2c) without evidence of distant metastases. Abdominal ultrasonography showed a 45 x 30 mm tumor in the head of the pancreas with a smooth surface and low-echogenicity, without pancreatic ductal dilatation (Fig. 2d). These imaging features led to the tentative diagnosis of a pancreatic NET although endoscopic findings suggested a duodenal GIST. Establishing the histological diagnosis is helpful to guide the operative approach. We decided not to perform EUS-FNA because of concern about possible recurrent bleeding. The patient underwent a laparotomy and the tumor was found between the third portion of duodenum and the pancreatic uncus (Fig. 3a). Curative resection necessitated resection of the papilla and part of the pancreas and a pylorus-preserving pancreaticoduodenectomy was performed. The resected specimen showed a 45 × 28 x 50 mm submucosal tumor, compressed and invading the uncus of the pancreas with clear resection margins (Fig. 3b, c). The tumor was comprised of spindle-shaped cells (Fig. 3d) on histologic evaluation without lymph node involvement. Immunohistochemical studies revealed that the tumor cells were positive for c-kit (Fig. 3e) and CD34 (Fig. 3f). The tumor was diagnosed as a duodenal GIST.

The patient had an uneventful hospital course. He was treated with adjuvant imatinib chemotherapy as an outpatient. Fourteen months postoperatively, there is no evidence of recurrence.

## 3 Discussion

GISTs are relatively common mesenchymal tumors which mostly occur in the stomach (60%–70%) and small intestine (25%–35%) [6], followed by the rectum, esophagus, omentum, and mesentery (<5%) [1]. Duodenal GISTs are rare lesions, constituting 30% of primary duodenal tumors and less than 5% of all GISTs [2]. These tumors mostly occur in second portion of the duodenum followed by the third, fourth and the first portions [11]. When a GIST is found in the second or third portion of the duodenum with extraluminal extension, it may be difficult to identify the origin of the mass.

Pancreatic NETs are uncommon and represent 1–2% of all pancreatic neoplasms [7]. Surgical resection with regional lymph node dissection is the only curative treatment for pancreatic NETs [12]. Both pancreatic NETs and duodenal GISTs have moderate to intense enhancement on CT scan [5,8], and it may be difficult to differentiate these tumors based only on diagnostic imaging criteria. Some reports have described that duodenal GISTs arising in the second or third portions of the duodenum might be incorrectly diagnosed as a pancreatic mass [3,13].

Cai PQ. et al, reported that arterial blood supply, intratumoral vasculature, and draining veins were all detected on enhanced CT images in 34 patients with duodenal GISTs [5]. Enhanced CT features of our patient were consistent with their criteria (Fig. 2a, b and c). Those findings on CT images can be clues to establish the diagnosis for duodenal GISTs.

GISTs are typically composed of spindle-shaped cells which are positive for c-kit, anoctamin1 and CD34 [14]. Histopathological and immunohistochemical studies are necessary to establish the precise diagnosis. EUS-FNA has been reported effective to establish the diagnosis of various lesions with a relatively low overall risk of complications (1.6%) [4]. However, gastrointestinal bleeding from a central ulceration is common in patients with a duodenal GISTs [1]. Gastrointestinal bleeding accounts for 30–40% of the clinical manifestations of GISTs, which is also the most morbid complication, often necessitating emergency surgery [6]. Duodenal GISTs may present with gastrointestinal bleeding more commonly [15] due to the abundant blood vessels in the submucosal layer in the duodenum compared with the digestive tract [16].

When considering EUS-FNA to differentiate a duodenal GIST from a pancreatic tumor, especially a lesion rich in feeding vessels, there is concern about post-procedure bleeding. The utility of EUS-FNA for the diagnosis of pancreatic masses has been reported [17]. Another report also described the sensitivity of EUS-FNA cytology was sufficient (84.4%) for the diagnosis of gastric GISTs but poor for tumors in the duodenum [18]. In consideration of the complications of EUS-FNA this was not performed for the present patient.

Due to the rarity of duodenal GISTs, the optimal surgical procedure has not yet been established [19]. Both segmental duodenectomy and pylorus-preserving pancreaticoduodenectomy are reported to be appropriate options to treat patients with duodenal GISTs [1]. There is no survival benefit for patients with a duodenal GIST treated with pancreaticoduodenectomy compared to limited resection [15], so minimal resection may be the preferred approach.

Lee, S. Y. et al, reported that 38% of patients with duodenal GISTs underwent pancreaticoduodenectomy [20]. They also described that patients who underwent pancreaticoduodenectomy tended to have larger tumors with the majority located in the second or third portions of the duodenum [20]. The tumor in the present patient located in the second portion measuring 5 cm, and pancreaticoduodenectomy was considered the best option for long term survival. We performed a pylorus-preserving pancreaticoduodenectomy in this patient.

When a lesion is found in the head of the pancreas or mesenteric side of the duodenum, the possibility that a duodenal GIST can be mimic a pancreatic tumor must be considered. The histological diagnosis should be established preoperatively when possible, if EUS-FNA can be performed safely. Intraoperative frozen section diagnosis may be helpful in some situations.

## 4 Conclusion

Duodenal GISTs are often misdiagnosed as a pancreatic head mass based on imaging criteria. The strategy for resection of pancreatic NETs and duodenal GISTs are different. Ideally the histological diagnosis can be established before surgery, but EUS-FNA has associated complications that must be considered.

## Conflicts of interest

All authors declare no conflicts of interests regarding the publication of this paper.

## Funding

All authors have no funding regarding this paper.

## Ethical approval

The need for ethical approval for this paper was waived by the committee of Jichi Medical University Hospital.

## Consent

Written informed consent was obtained from the patient for publication of this case report and accompanying images.

## Author contribution

All authors in this manuscript contributed to the interpretation of data, and drafting and writing of this manuscript. YF, SS, YK, AS, YK, HM and NS were engaged in patient’s care in her hospital coarse including surgery and endoscopy under the supervision of AL, HH and NS. AL helped in drafting the manuscript and interpretation of data. DM was engaged in pathological and immunohistochemical studies. All authors have read and approved this manuscript for publication.

## Registration of research studies

The name of registry is research registry, and the unique identifying number (UIN) we obtained is researchregistry4372.

## Guarantor

Dr. Sata, who is the president of Jichi medical university hospital, is the Guarantor.

## Provenance and peer review

Not commissioned, externally peer reviewed.
